# Subjective wellbeing in people living with dementia: exploring processes of multiple object handling sessions in a museum setting

**DOI:** 10.12688/wellcomeopenres.16819.2

**Published:** 2021-06-10

**Authors:** Paul M. Camic, Laura Dickens, Hannah Zeilig, Sarah Strohmaier

**Affiliations:** 1 Queen Square Institute of Neurology, University College London, London, UK; 2 Salomons Institute for Applied Psychology, Canterbury Christ Church University, Tunbridge Wells, Kent, UK; 3 London College of Fashion, University of the Arts, London, UK

**Keywords:** Dementia, Object handling, Museums, Wellbeing, Thematic analysis, Canterbury Wellbeing Scales

## Abstract

**Background:** Dementia care guidance highlights the importance of supporting people living with dementia to access engaging and meaningful activities to promote their quality of life. There is a growing evidence base for the efficacy of heritage settings and arts-based interventions to provide social prescribing opportunities to help support wellbeing in this population. This study extended previous research and explored the potential processes underlying this effect in multiple small group object handling sessions in a museum setting.

**Methods:** A mixed-methods design was used comprising a measure of subjective wellbeing and thematic analysis to explore in-the-moment session content across multiple sessions. Four people with dementia participated in three, one-hour group object handling sessions led by two facilitators.

**Results:** Pre-post wellbeing scores showed increases after each session though this was largely not significant. Qualitative findings provided more compelling results, however, and identified four key themes: facilitating, interest in exploring objects, active participation, and group collaboration; interpretations were made around the dynamic interaction of themes and subthemes over the course of three sessions.

**Conclusions:** This is the first study we are aware of that has taken an in-depth look at multiple museum-based group object handling sessions for people living with dementia. Findings offer ways to optimise object handling sessions for people with dementia by providing in-depth information about the processes involved across multiple object handling sessions facilitated by museum/heritage professionals in a museum setting. This has useful implications for community-based activities as part of dementia care planning and public health programming. The study contributes to a deeper understanding and elucidates the processes that enhance wellbeing for this population who participate in such sessions. It also helps to develop further theoretical understanding about why these types of activities are helpful in community-based dementia care. Limitations and implications for future research are discussed.

## Introduction

Although there are a range of symptoms, dementia is a syndrome often characterised by progressive decline in cognitive functioning, motivation, affective control and social behaviour ( Dementia UK, n.d.). It can impact adults at any age with the majority developing symptoms over the age of 65 years; it is an international public health priority, owing to its growing prevalence and associated social and economic challenges ( World Health Organisation, 2019). With no cure, promoting quality of life and wellbeing is central to supporting those living with a dementia ( Algar *et al*., 2014). Recognising the significant impact dementia has on both the person and their families and carers, dementia care guidance highlights the importance of enabling people to live independent and meaningful lives through supporting them to engage in meaningful activities ( National Collaborating Centre for Mental Health, 2018). Kitwood (1997) also advocates the importance of person-centred approaches to dementia care that recognise and maintain the personhood of people living with dementia (PLWD) in the face of cognitive decline. Therefore, increasing the availability and accessibility of meaningful and engaging interventions for this population is an important challenge ( Zeilig *et al*., 2014).

### Heritage and arts interventions

In the interest of supporting people to live well with dementia, it is important to explore opportunities for interventions that can increase quality of life and wellbeing in the wider community. Participative arts interventions (e.g. singing and music, dance, poetry and art-making, museum and art gallery activities) have become a growing area of interest due to their potential for positive outcomes for PLWD and carers ( All Party Parliamentary Group on Arts, Health and Wellbeing, 2017; Zeilig *et al*., 2014). In addition, arts and heritage environments (such as art galleries and museums) are often widely accessible and are recognised as having the potential to play an important role in health, wellbeing ( All-Party Parliamentary Group, 2017; Ander *et al*., 2013b) and public health, as non-stigmatising settings that promote learning and engagement ( Camic & Chatterjee, 2013), including for those affected by dementia ( Sharma & Lee, 2019). Museums also broadly provide important opportunities for social inclusion for older people experiencing isolation and loneliness ( Thomson *et al*., 2018; Todd *et al*., 2017). This has given rise to initiatives such as Museums on Prescription ( Veall *et al*., 2017) as part of the new overall social prescribing initiative supported by the National Health Service ( NHS England, 2019).

Researching art gallery and museum-based interventions for this population has suggested positive outcomes related to cognitive functioning ( Eekelaar *et al.,* 2012; Young *et al*., 2015) and wellbeing for those with early and middle stages of dementia ( Camic *et al*., 2019), as well as positive wellbeing outcomes for carers when participating in the same activity ( Johnson *et al*., 2017). Carers have also reported observing improvements in mood and confidence in their cared-for family members when participating alongside them during these interventions ( Eekelaar *et al*., 2012). In addition, research exploring the subjective experiences of those with dementia and carers participating in art gallery interventions has highlighted key enjoyable aspects of such interventions including cognitive stimulation, social connections and “self-esteem” ( Flatt *et al*., 2015).

### Object handling and wellbeing

Museum object handling is one such arts and heritage intervention that has a growing evidence base in promoting wellbeing. Camic *et al*. (2019) highlights that wellbeing is a multi-dimensional construct that has proven difficult to define in terms of theoretical consensus. They draw on the works of Dodge *et al.* (2012) and Huppert & So (2013) to consider the fluctuations in cognition, emotions and behaviour that PLWD can experience and conceptualise subjective wellbeing in dementia as a biopsychosocial process. This process involves “(1) various fluctuating internal states… that (2) are experienced in numerous different ways across the different types of dementia and where (3) the accessibility and use of external resources (e.g. stimulating activities that engage the senses combined with social support) can help mitigate internal states (challenges) and increase wellbeing” (p. 4). Camic (2010), investigating a non-clinical sample, proposes that discovering and exploring material objects can stimulate areas such as motivation, emotion and cognition, provoking curiosity, creativity and linking to personal memories and meanings. In addition, Solway *et al.* (2016) highlight the theoretical potential of the combination of multiple sense modalities, including the multidimensional sense of touch, to enhance memory, cognition and emotion.

Thomson *et al*. (2012) discuss theory relating to the interaction of the different sensory modalities (visual, verbal and touch) involved in object handling. They propose in addition to verbal and visual modalities, the tactile element in handling objects may further increase wellbeing through a “triple-coding model”. This builds on Paivio’s (1986) dual-coding theory of memory and cognition, where verbal and visual representations connect in working memory during encoding processes and are integrated with information in long-term memory. This also draws on Simmons’ (2006) proposal that this may also be enhanced by the “contiguity effect” ( Clark & Paivio, 1991), where the coordinated (rather than separate) presentation of verbal and visual information leads to improved performance. They also suggest that in line with Craik & Lockhart’s (1972) levels of processing model, the additional modality of touch may increase the “kinaesthetic experience” resulting in “deeper and more elaborate memory traces” being created (p. 76). This is especially relevant in the context of sensory impairment, which can occur in different types of dementia ( Alzheimer’s Society, 2016).

### Extant literature

Object handling has been shown to increase wellbeing and engagement across settings and client groups (see Solway *et al*., 2016 for a review of previous research in this area). Studies have also begun to investigate the features and processes underlying this effect. For example, Ander *et al.* (2013b) conducted a grounded theory study on a combination of group and one-to-one sessions and associated field notes and interviews, across a number of acute hospital wards, neurological rehabilitation units, an elderly psychiatric ward and an elderly care home. This focussed on the impact of sessions on wellbeing and described two key findings: the process of engagement (particularly in hospital patients due to the challenges of the setting, (e.g. a lack of stimulation and uncertainty) and expressions of wellbeing (including improved mood and confidence).

Paddon *et al*. (2014) used wellbeing measures and inductive and deductive thematic analysis to investigate the content of one-to-one object handling sessions in hospital patients (across older adult mental health, oncology and neurological rehabilitation wards). They explored processes relating to object engagement and facilitation and found sessions significantly improved wellbeing. “Thinking and meaning-making” was also discovered to be the most important aspect of the patient’s role in sessions, which they linked to promoting an increased capacity to cope with stressful events.

Elaborating on these findings, the review by Solway *et al*. (2016) suggests group processes, encompassing the use of museum artefacts, may occur that influence or enhance the outcomes and participants experiences of sessions. In line with this, Solway *et al.* (2015) used thematic analysis to explore open group object handling sessions in older people in a mental health ward. They identified five main themes: responding to object focussed questions, learning about objects and from each other, enjoyment, enrichment through touch and privilege, memories, personal associations and identity and imagination and storytelling, which they note reflect participants’ working in collaboration, interacting and sharing knowledge.

Only three studies to date that we are aware of have looked at the potential benefits of museum object handling sessions specifically for PLWD. Johnson *et al.* (2017) compared three small group activities: object handling, art-viewing, and a social refreshment break. They found significant increases in subjective wellbeing in both museum interventions for those with dementia and their carers, but not in the refreshment break. The authors reported a previous lack of evidence as to whether psychological benefits of arts interventions could be explained by social factors. They noted their study’s findings suggest benefits were not solely connected to the social element of the interventions. Camic *et al*. (2019) expanded on this study and found small group object handling sessions to increase subjective wellbeing in people with both early and moderate stages of dementia. Strohmaier *et al*. (2021a) examined previously collected data across multiple sites and types of interventions, including object handling, and found increases in subjective wellbeing across different arts-based interventions. All three studies utilised a subjective wellbeing measure that used the visual analogue-based Canterbury Wellbeing Scales (CWS; Camic *et al*., 2020), and emphasised the value of capturing “in-the-moment” changes which may otherwise be lost or where longer-term maintenance of benefits may not exist ( Camic *et al*., 2019).

In-the-moment activities and experiences for people with dementia, were identified in a mixed-methods study in an Australian art gallery setting ( MacPherson *et al*., 2009), which noted that benefits were not long lasting. The presence or absence of lasting impacts of interventions can overlook the meaning and importance of what being in the moment signifies for those living with a dementia. It seems less relevant to know if non-pharmacological interventions are long lasting in a population with a progressive, life threatening disease than to understand what types of moments bring engagement, enjoyment, interest, stimulation, comfort, challenge and confidence. More relevant questions for a dementia population include, How long do these moments last? What comes before and after them? Can different moments be linked together? ( Keady *et al*., 2020). 

Previous research has also highlighted the importance of the facilitator’s role and the qualities needed to engage and facilitate participation. This includes, having training in working with those living with dementia, group facilitation skills, providing knowledge of objects and questions to facilitate interactions with objects, and an interpersonal style that helps to create an atmosphere that supports PLWD to feel at ease and stimulates curiosity ( Camic *et al*., 2019). Understanding the ways in which facilitators can work to optimise sessions is an important consideration for museums, but also for other arts and heritage settings that value the potential of creative activities for this population.

### The present study

The literature to date provides promising support for the value of museum object handling sessions in improving wellbeing in a range of conditions, including dementia. An understanding of the mechanisms underlying these positive effects is also developing. However, there are no studies that we are aware of that explore the content and processes of group object handling sessions specifically for those with a dementia.

The present study was one component of the Created Out of Mind residency at the Wellcome Collection ( Brotherhood *et al*., 2017), and consequently develops the findings of Johnson *et al*. (2017), and Camic *et al.* (2019) to explore the *processes* that may contribute to an increase in quantitative measures of subjective wellbeing. Investigating these processes is important for public health planning of dementia care activities organised within the heritage sector ( Camic & Chatterjee, 2013) in order to better understand how museum object handing may promote subjective wellbeing and to optimise sessions for this population. 

### Aims, hypotheses and research questions

The present study had two aims: Firstly, to explore whether subjective wellbeing would increase in line with the previous studies outlined. The second and main aim of the study was to explore the processes within three facilitated small group object handling sessions in a museum setting in order to better understand the ways in which the sessions may be effective in promoting subjective wellbeing for this population.

The study was guided by the following research hypothesis and questions:
  H1: Mean scores of subjective in-the-moment wellbeing will increase post-OH sessions.
  Q1: What is the process of facilitation?
  Q2: What are the roles of material objects?
  Q3: What is the process of person-to-person interaction within the group?

## Methods

### Design and setting

This study adopted a mixed-methods design. This comprised a freely available, quantitative pre-post self-report measure of wellbeing (CWS; Camic *et al.,* 2020) across each of three sessions, and qualitative thematic analysis of continuous audio and video recorded content from three group object handling sessions.

The study took place at the Wellcome Collection, a free, publicly accessible specialist museum in central London near public transportation. The museum’s focus is to connect science, medicine, health and the arts and this is reflected in its collections, special exhibitions, public engagement, and lecture programme. The object handing sessions took place in a well-lit, comfortable conference room with windows on a high floor. Facilitators and participants were seated around a rectangular table and close enough to allow for the easy passing of objects. The physical environment of the room was considered and the decision to hold the sessions in a private space rather than a gallery room was made to allow quiet for listening, fewer distractions to support engagement and confidentiality for audio recording. The research was part of a larger, two-year research programme, Created Out of Mind, that sought to challenge and shape perceptions and understanding of dementias through science and the creative arts. 

### Measures

The Canterbury Wellbeing Scales is an easy-to-complete subjective measure of wellbeing using visual analogue-style scales ( EuroQoL Group, 1990), with good reliability ( Camic *et al*., 2019; Johnson *et al.,* 2017; Strohmaier *et al.,* 2021a) in a dementia population. It was specifically developed to look at dimensions of in-the-moment wellbeing relevant to both people with dementia and their carers, and comprises five subscales (Happy/Sad, Well/Unwell, Interested/Bored, Confident/Not Confident and Optimistic/Not Optimistic). Each scale is presented vertically from 0 to 100 and participants are asked to place a mark to show how they are feeling in the present moment. Scores for each subscale are also summed for a composite wellbeing score.

***Stakeholder involvement.*** Those living with dementia and carers were part of the initial development of the CWS. Their involvement included discussion of the scale’s variables to be measured, how many subscales to include, font size, use of face images at high, mid and low points, and ease of understanding the directions. Additional feedback from participants in previous studies ( Camic *et al*., 2019; Johnson *et al*., 2017) was used to determine the number of sessions and the number of objects used in the present study.

### Thematic analysis

Thematic analysis, a well-known and frequently cited qualitative methodology, was used to analyse session transcripts and field observation notes. This methodology allows for a close inspection of data in order to identify patterns and themes within and across sessions, thus providing an in-depth investigation of the phenomena at hand ( Braun & Clarke, 2006). Clarke & Braun (2018) emphasise thematic analysis is an umbrella term describing a range of different approaches which vary in their philosophical underpinnings and procedure for analysis. A “coding reliability” approach ( Clarke & Braun, 2018, p.108) was adopted in this study in line with Boyatzis (1998), utilising a structured approach to generating codes and themes to improve their accuracy and reliability. This study was underpinned by a critical realist epistemological approach which posits the existence of an objective world, independent of human language and perception, whilst also acknowledging that this world is in part made up of subjective interpretations that influence how it is experienced and perceived ( O’Mahoney & Vincent, 2014).

### Ethical considerations

The study was granted ethical approval by a Canterbury Christ Church University ethics panel (075\17-18). The research adhered to the British Psychological Society’s Code of Human Research Ethics (2014) and the UK Data Protection Act (2018).

### Participants

To help further situate the sample and act as a screening tool, the brief version of the mini mental state examination (MMSE – 2 BV; Folstein *et al*., 2010) was administered by LD after written informed consent had been obtained. The clinical dementia rating (CDR) scale ( Morris, 1997) was completed by a family member; CDR scoring ranges from 0 (no impairment) to 3.0 (severe impairment) across six categories. Although all participants were classified as being in the mild impairment stages of their respective dementia diagnoses, designating impairment levels in dementias is not necessarily precise and ability to consent should not be assumed based solely of these assessments. In particular, the MMSE is known to underestimate cognitive ability and the CDR has only been normed on people with an Alzheimer’s dementia diagnosis. Two participants were deemed to have capacity to consent to participation ( Mental Capacity Act 2005, 2007) and two had a spouse act as a proxy to support that participation was in line with the participant’s wishes. All participants attended all three object handling sessions. Two female museum visitor experience guides, experienced in handling artefacts, facilitated the object handling sessions.

***Recruitment.*** Across a range of local dementia settings and charities, posters describing the study were emailed and displayed both online and in day centres, waiting rooms and at a dementia involvement group. In addition, permission was granted by Join Dementia Research, a dementia research database, for recruitment. Eligibility for the study included being aged 50 years and above, having a confirmed dementia diagnosis in the mild-to-moderate stage, being able to commit to the three group sessions, and having no significant co-morbid psychiatric or health conditions that could impede group participation.

Those who expressed an interest in the study attended a pre-study meeting in order to confirm eligibility, read and discuss the participant information document, answer any questions, gain informed consent, complete the MMSE-2 BV and the CDR. Participants were asked to describe the study in their own words to assess capacity to consent. Three attended with their spouse and one alone. This also provided an orientation to the space where the sessions would take place. One participant who required a proxy for the consent process was asked to bring someone with them who would remain in the museum for all three sessions, and could be contacted if needed.

### Object handling sessions

Participants attended three one-hour object handling sessions over three consecutive weeks at the same day, time and location in order to create consistency and a sense of familiarity. The second session also involved smelling four spices contained in small glass bottles (passed one at a time to participants) that were part of the museum’s collection. Olfactory experiences in dementia care have been shown to be potentially engaging experiences that can evoke personal responses and reflection. Three sessions were chosen based on the design of a previous arts intervention study by Eekelaar *et al*. (2012) and as a time frame that allowed for multiple sessions to maximise data collection for each participant, without burdening participants. It was also decided, in consultation with museum staff, that three one-hour sessions would have ecological validly for a museum environment. This built on the opportunity to assess the feasibility of running a series of sessions within this population where the person with dementia may require someone to accompany them on the journey to and from the venue. The total length of the sessions was approximately 2 hours to allow time either side for participants to arrive and have refreshments, engage in general conversation with each other and the researchers, and to orientate themselves to the setting. The CWS was completed immediately before and after each session for a total of six time points across three sessions.

Sessions were guided by a protocol ( Dickens *et al.*, 2021) that was created in collaboration between the researchers and facilitators and informed by previous object-handling feedback and research ( Ander *et al*., 2013a; Camic *et al*., 2019; Camic *et al*., 2018; Johnson *et al*., 2017). Facilitators welcomed participants at the beginning of each session passing around one object at a time and encouraged touching and generating discussion through asking a range of sample prompts and questions to encourage participation and exploration before sharing information about each object. Questions were used flexibly in an open, dialogic way within the sessions based on the interaction of participants. A total of 20 objects were used across the sessions; 14 objects came from the museum’s collections including 4 spices, while others were contributed by PC. Six to nine objects were used in each session. Objects were jointly decided upon by the facilitators and researchers and selected to be novel and diverse in their cultural, historical and sensory qualities in order to maximize variety, interest and opportunities for exploration that did not rely on previous memory or knowledge. The study’s protocol, including sample questions, photos and descriptions of the objects used in each session are available as extended open access files ( Dickens *et al.*, 2021).

 Two 360-degree Fly™ cameras (360fly, Canonsburg, PA, United States), providing an uninterrupted 360-degree recording of group interaction, were used to record the verbal and visual content of sessions. About the size of a tennis ball, this device is unobtrusive and did not appear to distract from the objects or group interaction. An additional audio recording device was also used as a backup. Sessions were led by two Wellcome Collection facilitators who were trained in working with people living with dementia. LD, PC and HZ observed all sessions unobtrusively from the back of the room but did not take part. 

A handout was provided after each session ( Dickens *et al.*, 2021) consisting of pictures and information on the objects explored and the time and date of the next session as a memory prompt. At the end of the final session, the group curated a display of all the objects used in the study that was available for public viewing for one month. At the conclusion of the study, shopping vouchers (£30) were given to thank people for taking part.

### Quantitative data analysis

To determine whether there were significant changes in CWS scores before and after each session as well as between baseline (pre-session one) and post-intervention (post-session three), session-by-session and baseline to post-intervention repeated measures pairwise t-tests were completed using SPSS version 24. These were completed for each of the five subscales as well as the composite CWS and analysed by SS.

### Qualitative data analysis

Audio content from the entirety of the three object handling sessions was transcribed and subsequently coded using software package NVivo 12. Following Braun & Clarke’s (2006) guidelines for thematic analysis, LD initially viewed all video recorded sessions and read session transcripts in full. Full transcripts from the three object handling sessions were then coded (approximately 200 pages of text) for both semantic and latent themes. Semantic themes captured how sessions were facilitated and how objects were explored. Latent themes captured interactions and processes within the group. Video data were consulted to clarify understanding of the transcripts for accurate coding. In line with a “coding reliability” approach ( Clarke & Braun, 2018, p. 108), a codebook ( Boyatzis, 1998) was developed across the three sessions as codes were generated, to capture codes and their descriptions. This was revised and refined to collapse any codes that were too similar or not pertinent to the research questions. Codes were also further broken down where this provided additional relevant information. Through this process, a final codebook of the three sessions was developed. Initial themes were subsequently developed and refined based on these codes and subthemes were identified. All codes (and subsequently developed themes) were discussed in detail with LD, PC and HZ, examining supporting quotes throughout, to improve the reliability and validity of the analysis. In addition, discussions also took place with two other colleagues, both at the stage of code development and theme and subtheme development. 

*Quality assurance*. Meyrick’s (2006) guiding framework for rating the quality of qualitative research for transparency and systematicity was consulted to inform the process at each stage. Feedback gathered from PLWD and carers in previous projects was used to inform the design of the sessions. This is in line with quality assurance ( Weinstein, 2006) and the National Institute for Health Care Excellence (2019) quality standard statement on providing activities to promote wellbeing through discussing with PLWD their needs and preferences to inform these. In addition, a reflective research journal was kept by LD and HZ throughout the study as a way of exploring subjectivity and possible biases, which was discussed with PC on an ongoing basis. For example, some of the issues discussed included the researchers’ own feelings of interest towards the objects, positivity about the potential benefits of object handling, and the need to remain open to possible positive and negative participant experiences within the sessions.

## Results

### Participant characteristics

Data were gathered from four white British participants diagnosed with a dementia ( Table 1), all of whom were living in the community, three with a spouse and one alone. A further four potential participants expressed interest in the study but two withdrew their participation due to diary conflicts with the session dates; two did not give a reason. 

**Table 1.** Participant demographic information.

Participant	Age	Gender	Type of dementia	MMSE-2 BV	CDR
1	64	Male	Alzheimer's	12	0.5
2	86	Female	Alzheimer's	14	0.5
3	65	Male	Frontotemporal-familial variant	13	1.0
4	61	Male	Frontotemporal-behavioural variant	11	1.0

*Note.* MMSE-2 BV = Mini Mental State Examination 2nd edition: brief version. This is out of a total score of 16 with lower scores indicating cognitive impairment. CDR = Clinical Dementia Rating scale. This is out of a total score of 3 (0 = no impairment to 3.0 = severe impairment).

### Subjective wellbeing scores

**Figure 1.**
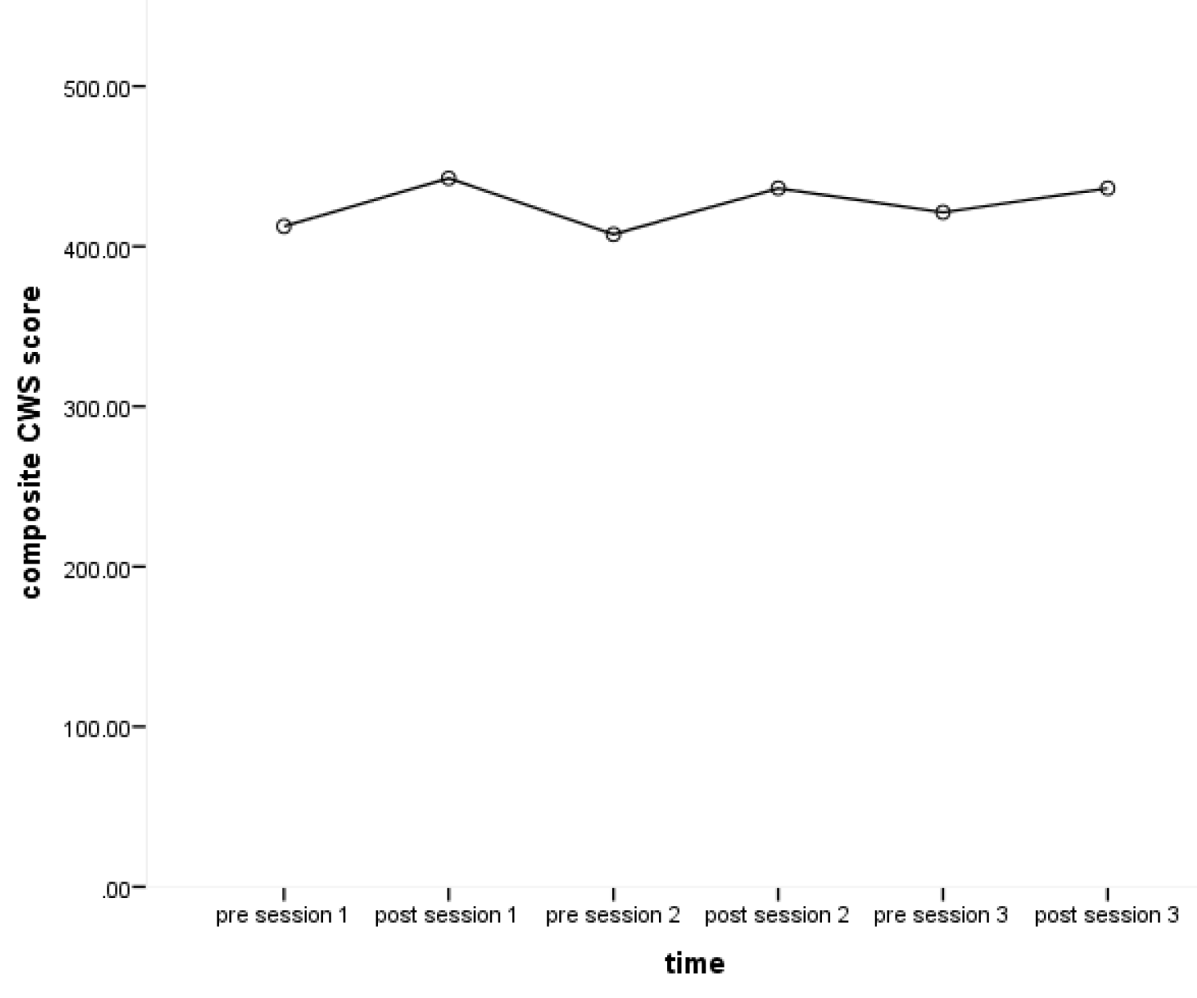
Change of composite CWS (Canterbury Wellbeing Scales) scores pre- to post-sessions over the course of the intervention.

Mean pre-post CWS scores for each of the five subscales (Happy/Sad, Well/Unwell, Interested/Bored, Confident/Not Confident and Optimistic/Not Optimistic) and composite scores of all subscales were calculated for each object handling session ( Strohmaier *et al.,* 2021a). Scores on the CWS, including each of the five subscales as well as the composite scale, increased after each session (from pre- to post-session) as well as from baseline (pre-session one) to post-intervention (post session three). Figure 1 shows the change of composite CWS scores pre- to post-sessions over the course of the intervention showing greater CWS scores at post-session compared to pre-sessions. This increase in self-reported wellbeing post- sessions for all subscales, when compared with pre-session ratings, compares favourably to previous studies ( Camic *et al*., 2019; Johnson *et al*., 2017; Strohmaier *et al*., 2021a). Table 2 shows pre to post change before and after each session as well as for pre- to post-intervention. Overall, participants scored their wellbeing highly after each session, with an average post-session composite CWS score of 438.33 out of 500. A statistically significant increase was found for the Interested-Bored subscale after session two ( *M* = 11.25; *p* = .037). However, although some pre-to-post changes approached significance, the majority of pre-to-post change scores were not significant. This is likely a Type II error due to the very small sample ( *N* = 4) and with a larger sample size, may have been significant.

**Table 2.** Group mean pre-post subjective wellbeing change scores.

CWS Subscale/ composite score	Session 1	Session 2	Session 3	Pre-post change
Composite score	+30	+28.75	+15	+23.75
Happy	+7.5	+3.75	+5	+6.25
Well	+ 10	+5	+2.5	+3.75
Interested	+8.75	+11.25 *	+2.5	+7.5
Confident	+1.25	+7.5	+2.5	+2.5
Optimistic	+2.5	+1.25	+2.5	+3.75

*Note.* CWS = Canterbury wellbeing scales. Composite score = sum of the subscales. Subscales are scored from 0 - 100 and the composite score from 0 – 500; pre-post change = pre-session 1 to post session 3 change; * *p*<.05.

Change scores ranged from an increase of 1.25 to 11.25 points for individual subscales (out of a possible score of 100). The greatest subscale change score at session 1 was for the Well subscale, in session 2 the Interested subscale and at session 3 the Happy subsale. For the composite score, average change scores ranged from an increase of 30 points at session 1 to 15 at session 3 (out of a possible score of 500). These change scores are in the same direction as those reported by Camic *et al*. (2019) who used a larger sample size (n = 80) and found participant composite scores to increase by an average of 57.81 points and Johnson *et al.* (2017) who found an overall increase of 30.29 and 39.74 points (n = 36).

### Overview of themes

**Figure 2.**
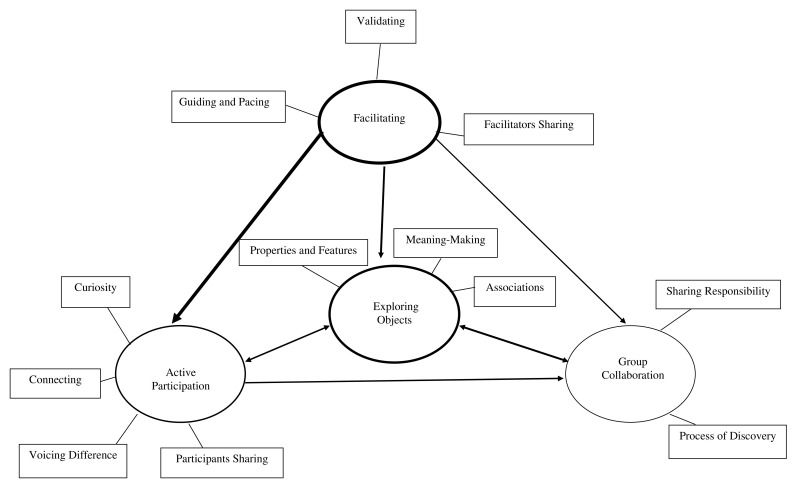
Thematic map. *Note*. Arrows signify the direction of the relationship between themes. The thickness of the line depicts the strength of this relationship, with a thicker line representing a stronger relationship.

Data were analysed using an inductive thematic approach to identify themes in order to address three research questions: Q1: What is the process of facilitation? Q2: What are the roles of material objects? Q3: What is the process of person-to-person interaction within the group? The final thematic map ( Figure 2) and the themes and subthemes with example codes and supporting quotes are outlined in Table 3.

**Table 3.** Summary of themes.

Theme	Subtheme	Relevant codes	Supporting quotes
Facilitating	Guiding and pacing	F asking questions (exploration; memories, existing knowledge or personal experience; opinions and preferences). F direction or instruction - prompting F changing topic or introducing new idea F providing information F answering questions	F1: “Hmm, do you want to have a look and just pass it around? How does it feel? It’s pretty heavy.” F2: “And it’s used for you know if you had a beautiful wooden floor and you didn’t want that to get scratched by your legs of your chairs and tables.” F2: “What would you use it for, do you think?”
	Validating	F bringing people in F validating or encouraging to P F responding to P	F2: “So have people have seen them before? So it sounds like you have. Does anyone else? Have you not seen them?” F1: “It doesn’t look edible, yeah, I agree, especially when you hold it” P4: “That’s for cooking”, F2: “You’d use it in the kitchen”
	Facilitators sharing	F not knowing F sharing opinions F sharing personal information Humour	F2: “I think that’s why I don’t, for years I didn’t like it, because I think we were given it as children erm, and it put me off it. But I like it now.” F1: “I think as a sense, smell in general is quite under erm represented and we don’t really talk about, we’re quite like visual as as a society. So maybe we do smell, but we’re not aware of it.” F2: “I had absolutely no idea actually what that was for. And then I, last night, I was looking at something my father had, which is a little elephant, a little iron elephant with holes in it. And that’s very clearly for incense, it’s got a hole in the bottom and you put a a burning comb into it and it comes up through the holes.”
Active Participation	Curiosity	P asking questions P guessing and hypothesising	P1: “I think it’s probably something medieval” P4: “I think it’s just a paperweight.” P3: “Is it that they thought of handling it maybe it would be get the bit of iron into their system somehow?”
	Connecting	P responding to F P asking P question P clarifying P agreement with another P Ps agreeing or reaching consensus P short-phrase engagement or contribution Ps responding to each other P changes or introduces new topic or moves conversation on	P1: “I can’t believe that that’s 18th century, can you?” P4: “I believe that they could be used in that time, but I don’t know how they would make it.” P3: “I didn’t hear what you said.” P4: “I said it’s looking at me, it’s got a pupil, it’s got an iris, and it’s the white.” P3: “It’s funny, I was thinking just the same, it’s like an eye face, like that.” P2: “Really?” P3: “Oh god, ask [name]. Get it over and done with.”
	Voicing difference	P disagreeing with or challenging others	P1: “I’m not quite sure that is true actually” P1: “Yeah, the bit, I mean I quite like the beads but erm” P3: “I think the beads are a distraction.” F1: “I’m sure you can use it as an ashtray if you want.” P3: “Yes, multifaceted.” P4: “No, because there’s nowhere to put the cigarette.”
	Participants sharing	P providing explanations or reasoning P uncertainty, not knowing or forgetting P sharing opinions and preferences P sharing personal information and stories P sharing personal knowledge Humour	P2: “Gosh, it’s not too heavy, it’s a bit, it’s obviously quite intriguing. Amazing.” P3: “I remember my auntie used to make apple pie with a lot of that. I really didn’t like it actually. I couldn’t really say anything, so” (all laugh). P4: “I got it because of the, the wood was very interesting and then I couldn’t bring it back to England and I didn’t see it for seven months because it was travelling by itself. And when I got, when I opened it, I sort of felt I must respect it.”
Exploring objects	Properties and features	Details or features Shape Material Fragility Visual properties Size Weight Orientation Smell Taste Touch or texture	P4: “It smells of reed.” P2: “different surface on the inside here, smooth surface on the outside” F1: “And what is the other one, and what shape is the other one?” P4: “Is it wood or is it ceramic?”
	Meaning- making	Identity or function/purpose Origin Age Meaning Ownership Quality Authenticity Production Condition Usefulness of object Monetary value Practicality Object skill or appreciation Danger	P2: “It’s very good quality.” P4: “You’d have to be very careful where you hung them, because if you’ve got lights through them, they could cause a fire.” F1: “Exactly, I think it’s because you’re just wealthy and you want to show off.”
	Associations	It’s like… Associations beyond the physical object	F1: “But I mean you’re, you’re right to associate smells with with rituals, that’s absolutely is been happening throughout different religions.” P4: “It looks like a tooth; P4: It’s got the root and then the little tooth.” F2: “Yeah, it looks like a face.”
Group collaboration	Sharing responsibility	Remembering – recapping what has been discussed Humour Sharing Introducing new idea/moving on	F2: “Somebody said drink, which was along the right lines.” P3: “I think he came up with that one.” F1: “Have you ever had any kind of things that I don’t know when you were kids your mother gave you? I mean like you know, in [name], my mum was giving me like honey and and lemon and and this type of things instead of I don’t know, paracetamol maybe.” P4: “Well not as a child, but when we were on expeditions, we used to have coco, because if you got a tummy bug, somehow it stops diarrhoea and things.” F2: “Does anybody else have got any idea what these could be? It’s related to people going ‘ouch’.” P4: “So you distract them, you give them that to play with and then you stick the needle in them” (all laugh).
	Process of discovery	P and F interactive learning Process of discovery and problem solving Co-curating	P4: “I think it’s either a key to a a castle or something or it’s a thing for turning off the main water.” P3: “It is.” P2: “I can see it.” P4: “I don’t know.” F2: “What do you two think?” P1: “I haven’t a clue.” P3: “I I thought it was a handle you know, but it probably isn’t, because that would be too simple, or a door knock. I don’t know.” P2: “Yeah, quite, it would be.” P4: “Quite difficult to carve so small, just because I thought at first, oh well, there’s much more work, but one the other hand, to do something as small as that and to make the hole.” P3: “Yes that’s right, very small.” P4: “And how did they make the holes, did they burn them in?” P3: “I don’t know, I hadn’t thought of that one.” P4: “Does it look as though they’re burnt in?” P1: “I don’t think you would’ve done that.” P2: “No, I wouldn’t think so.” F1: “Someone said carved, I think.” P1: “Yeah, I thought.” P2: “Oh it must be carved, I think.” P4: “Yeah, but they’re so perfectly round.” P1: “And the other one is like a snail.” F2: “Yeah.” P4: “So why a snail I don’t know. It certainly is a snail.” P1: “I don’t know. Well is it a snail? It’s quite an original snail.” P2: “Goodness curious.” P4: “Yeah, because look, it’s got the little horn.” P1: “It’s more of marine-type shellfish thing” P2: “Amazing.” P4: “No, but it’s got the horns on it. Can you see the horn?”

*Note.* P=Participant(s); F=Facilitator(s).

The thematic map displays the themes and subthemes identified within and across the group sessions. Arrows and lines depict the dynamic interactions between themes, with thicker lines representing a stronger relationship. *Facilitating* conditions created by the contributions of the facilitators, led to group *members actively participating* in expressing themselves and interacting in a variety of ways, *exploring objects* from a range of perspectives. This led to a sense of group cohesion and *group collaboration* (including both participants and facilitators) in the further exploration of objects. Objects appeared to provide a shared focus, acting as a vehicle through which these processes took place.

### Themes

***Facilitating***

This theme relates to the process of facilitation during the object handing sessions and comprises three subthemes: *guiding and pacing, validating* and sharing.

***Guiding and pacing*** reflects the direct guidance facilitators expressed to encourage exploration and learning. This included direct questions to participants to prompt or further exploration (F2: “Any idea where, what part of the world it might come from?”), relating to participants own experiences or knowledge (F1: “Where else is there is turmeric usually, in which food, which type of cuisine?”), eliciting opinions or preferences (F2: “Would you like one of those, would you have one in your house?”) and instructions or changes in topic, which also appeared to help structure the sessions (F2: “Well, let’s look at another object, very different”). This also included information provided by facilitators around objects (F2: “So this is a lucky iron fish and it was originally manufactured in Cambodia”) or to pace sessions by prompting further group discussion before sharing knowledge about an object (F2: “Actually, let’s work out first how old it is...”).

***Validating*** describes the action taken by facilitators to acknowledge what participants have said, responding to and encouraging their contributions (F2: “So it does have a function. I mean you’re right, some of it is luck, it’s to do with good luck”). This also included efforts to reach out directly to participants who may have been quieter in the session to support their participation, valuing all members of the group (F1: “Would you like to pass it on to [name] and see what he thinks”).

***Sharing*** referred to facilitators self-disclosing within the sessions, such as expressing not knowing (F2: “We were puzzled because it’s not an object from the museum, it’s from [name], he’s got an interesting collection of objects and we were totally baffled by it”), sharing personal information and their opinions around objects (F2: “It’s got a great fragrance.”) and using humour (F2: “So it wouldn’t have gin in it (laughs)?”).

Overall, *facilitating* may have contributed to a sense of equal status in the group between facilitators and participants and enabled safe and respecting conditions for participation.

***Active participation.*** This theme relates to the contributions of participants within the object handing sessions and is made up of the subthemes: *curiosity, connecting, voicing difference* and *sharing*. Independence and the confidence to actively participate within the sessions may have been enabled by the conditions created by the process of facilitation.

***Curiosity*** relates to the participants’ stance in the sessions around the objects and associated group discussion in asking questions (P3: “Is it an animal, standing, or is it a …?”) and guessing and hypothesising (P4: “I think it could be a very, very early mirror.”). The latter formed the most prominent part of the sessions towards exploring and discovering more about objects. This may reflect the sense of safety created in the group that allowed participants to take risks and guess without the fear that may be associated with giving an incorrect response.

***Connecting*** describes the ways participants communicated with other participants and facilitators within the sessions. This was often in the form of asking questions, which may have been directed at other participants, facilitators or the wider group (P3: “Which country was that in then? Where was that?”), to clarify what had been said (P3: “So that would be, you’d put the leg into that”), or responding more generally. This is inclusive of all participants and their own personal patterns of communication. For example, one group member generally communicated using shorter phrases (P2: “Amazing; Remarkable; Gosh”) than other participants, however the frequency of their contributions suggested they were engaged. Another group member more frequently took on the role of changing topic or moving the group on (P1: “Okay, what are we going to look at next?”). This may reflect participants’ idiosyncratic personalities and the roles they take up in groups, or possibly the impact of dementia on communication skills.

***Voicing difference*** demonstrates participants’ ability to express differing opinions, disagree with and challenge others in the group (P1: “I just can’t believe you’d stand a table in a glass, that wouldn’t, that doesn’t make sense to me.”). This occurred frequently during discussion as hypotheses were generated and appeared to be tolerated by and even drive the group in making further hypotheses.

***Sharing*** refers to participants self-disclosing within the session. This may relate to the sharing by facilitators, which may have been enabling for participants to feel able to share. Participants expressed not knowing (P2: “I don’t know what you’d use it for”), shared personal opinions (P1: “I think this is fantastic.”), personal stories and experiences (P3: “Well, I was in Paris, when I was about 18, 16, I don’t know. And erm and I bought a couple, not same as [name] but you know, one of them flea markets and black little figures like that.”) and humour (P3: “Get that out and hope it doesn’t mess up the rest of the stew”. (All laugh)).

### Exploring objects

This theme, comprising three subthemes: *properties and features, meaning-making* and *associations*, refers to how objects were explored through discussion in the OH sessions by both participants and facilitators. The number of these reflects the “multifaceted” (P3) nature of many of the objects. The fact that many of the objects were items participants, and in some cases the facilitators, did not know much about, appeared to allow them to be explored from many different angels. As such, the objects may have acted as a vehicle for a wide range of interaction within the group.

***Properties and features*** of objects refer to discussion about the physical objects themselves. For example, their weight or material (P1: “It’s quite heavy. I think its iron, is it iron?”), smell (P3: “It smells kind of like iron, that kind of unpleasant kind of smell.”), and decorative features (P4: “It’s got a lovely pattern on the bottom”).

***Meaning-making*** describes how group members responded to and made sense of objects beyond their physical properties. There was much discussion around the potential identity and function of the objects (P4: “I would now put moth, anti-moth things in it (laughs) but I don’t think that’s what it was for”) and their age (P1: “I mean to me it looks 20th century”). This subtheme also included discussion around whether objects were authentic (F2: “Is it real, I’m going to ask, is it real?”), their origin (P3: “It’s from China is it?”), and what they may represent (P4: “The eye, the eye, the eyes are very distinctive and I think that would tell you what tribe, if you knew enough about it.”).

***Associations*** captures the links that were made beyond the objects. This included likening objects to other things (P3: “It looks like a face to me, I mean you know, I just see it like that.”) and conversations that led on from the discussion of objects. For example, during a conversation about an iron fish, discussion led to the role of iron in diet (F1: “Yeah, especially I get, very you know more sensitive groups like pregnant women for example if they don’t, it can be quite dangerous if they don’t have enough iron, yeah.”) and when exploring a glass furniture leg protector (P4: “Because it’s not blown, you don’t chip at glass. So, when did press glass come in? Because that’s press glass, but when?”). This also links to personal stories that were shared, for example when using spices in the session (P4: “Hmm, I make French toast with cinnamon.”).

***Group collaboration*** This theme relates to the process of the group coming together as a whole within the object handing sessions and comprises the subthemes: *sharing responsibility* and process of discovery.

***Sharing responsibility*** describes the finding that both facilitators and group members came to share, as reflected within the themes of *facilitating* and *active participation*. This participation of sharing stories, using humour and moving the group on as previously reported, as well as recapping what had been discussed (F1: “I think you said cinnamon.”) appeared to reflect a shared responsibility for group participation. This may have contributed to a sense of equal status in the group and group cohesion.

***Process of discovery*** reflects the process through which members built on each other’s ideas. This described the learning of new information (P1: “What’s divination?”, F2: “Erm, well sort of trying to see the future, trying to work out what’s going to happen.”), sharing different ideas and problem solving around objects (P1: “I think it is an ashtray isn’t it?”, P4: “No, because it’s not big enough to put a cigarette.”, P2: “Not there”, P1: “No, that’s true, but if you turn it around that way.”, P4: “But still, there’s nowhere to put it, the cigarette.”) and the co-curation of a display case in the final session (P3: “Is there any, can we use this oval space?”, F: “Absolutely. There’s this piece here if you want to put that somewhere?”, P4: “No, no, it’s too similar to that, isn’t that?”).

### Multiple sessions

Researchers (LD, PC, HZ) looked at the final frequency of each code across each of the three sessions as well as the identified themes, to explore whether any clear changes or patterns could be identified across the sessions. Some fluctuation in the frequency of codes was observed with the varying topics of conversation around different objects. However, no clear changes or patterns were found, suggesting the frequency of the codes and the overall themes were relatively stable across each of the three sessions. Across all three sessions, the most frequently recorded codes were those relating to *exploring objects* (including both participants and facilitators and in particular around identity and function) and codes relating to *facilitating* and *active participation*: asking questions (both facilitators and members), participants guessing and hypothesising, participants sharing opinions and preferences and facilitators providing information.

### Summary of findings

Pre-post CWS scores may suggest an overall increase in participants’ self-reported wellbeing after object handling sessions.

The identified themes generated from the verbal content of sessions suggest that wellbeing may have been increased through the process of facilitation (facilitators *guiding and pacing*, *validating* and *sharing* to encourage participation), which may have empowered participants to have *actively participate* in expressing themselves ( *sharing curiosities and stories, making connections* and *voicing different opinions*). This led to *group collaboration*, between participants and facilitators, *sharing responsibility* for the group discussion and in building on each other’s ideas to come to *discover* more about an object together. The objects appeared to provide a shared focus within the group through which these processes took place, demonstrated by the many perspectives from which they were explored ( *exploring objects: properties and features, meaning-making and associations*). It is possible these processes impacted on participants’ experiences of feeling happy, well, interested, confident and optimistic, as reflected in the CWS.

## Discussion

The most important aspect of this study is providing the first detailed account that we are aware of, describing the in-the-moment processes occurring within museum object handling sessions in relation to their facilitation, the roles of material objects, and person-to-person interactions. This contributes towards a greater understanding of the ways in which this activity may be effective in promoting in-the-moment subjective wellbeing for people with dementia.

### Subjective wellbeing

Statistical analysis of the CWS revealed that there was an overall increase in scores post sessions, which is in line with previous research findings in a dementia population (e.g. Strohmaier *et al.,* 2021a). In particular, the greatest and only significant increase was found post session 2 for the Interested/Bored subscale with participants showing a significant increase in interest after having participated in session 2. This session involved different olfactory experiences, in addition to visual and tactile ones. Objects included a sandalwood elephant, a woven straw basket, black peppercorns, cinnamon sticks, cloves, and turmeric ( Dickens *et al*., 2021). Each of these objects invited engagement through three senses, sight, touch and scent, which may have increased interest in the activity. Olfactory experiences in dementia care ( Burns *et al.,* 2002) have generally revealed positive responses across different stimuli (e.g. Jakob & Collier, 2014; Tischler & Clapp, 2019) and should be considered when planning object handling sessions.

Verbal engagement was pronounced and involved a good deal of discussion, questioning and sharing of personal stories associated with the fragrances and objects. The frequency of the codes “facilitators bringing participants into conversations” , “participants sharing personal information and stories” and “participants responding to each other” were higher in session 2 relative to sessions 1 and 3. The frequency was also lower in session 2 for “facilitators providing information”. These may also have been associated with increased interest. However, frequency data was not a central aim of the analysis and can only provide a tentative exploration into this result.

### Qualitative analysis

The theme *facilitating* described how facilitators worked to create an atmosphere that enabled participants to feel at ease and supported participation. These are important factors for promoting engagement ( Camic *et al*., 2019; Todd *et al*., 2017). In providing a closer analysis of facilitators’ contributions, which make up the process of creating such an environment, this study offers important insights for training museum facilitators. For example, using humour, sharing personal experiences, being open about not knowing all the information about an object and prioritising exploration, in order to create a sense of equal status within the group. This can empower those with dementias in non-clinical settings to express themselves and share their own thoughts and ideas.

The theme *exploring objects* reflected the many different perspectives from which objects were explored and discussed. The number and range of different and novel objects used may have supported this, providing increased opportunities for multisensory and kinaesthetic experiences. This may reflect the triple-coding model proposed by Thomson *et al.* (2012) in that participation was increased through the combination of sensory stimulation, including touch in handling the objects, which may have been particularly beneficial in the face of other possible dementia-related difficulties. Handling the objects may have enabled continued and valued participation in a way that only visual and verbal stimulation alone may not have afforded. This may also have empowered participants by meeting their different abilities and needs within the group. Thus, the exploration of objects also links to the theme of *active participation*, relevant to the role of the object in providing a joint focus within the group, for promoting participation, self-expression and interactions with others. In relation to art therapy, Isserow (2008) describes the triangular relationship between an art object, therapist and client in which the joint attention of the therapist and client is directed at the art object. This underpins the therapeutic work in promoting a shared experience to share feelings and meaning-making opportunities.

*Active participation* was the most prominent theme in relation to group members and has some overlap with several themes identified in an older people’s mental health setting by Solway *et al*. (2015). For example, “imagination and storytelling” in participants sharing personal stories and “learning about objects, learning from each other” in asking questions and sharing opinions. *Active participation* appeared to be a particularly important finding given PLWD can often be disempowered both due to cognitive impairments and the attitudes and actions of others around them and stigma in wider society ( Kitwood, 1997). As part of this theme, participants displayed a confidence and independence in being able to direct questions to and challenge each other and share different ideas, which encouraged *group collaboration*. As outlined by others, this reflects the potential added benefits that can come from the social interactions within the groups ( Zeilig *et al*., 2019). Research has shown that engaging in activities with others in heritage settings can reduce isolation and provide a sense of “normality” for PLWD and caregivers through taking part in activities in the community as they may have done before the onset of dementia ( Sharma & Lee, 2019).

Paddon *et al*. (2014) reported that certain “features” identified within their thematic analysis appeared specific to a participant or facilitator, but that “interactional aspects of the sessions strongly implied that features were interlinked” (p. 37). This was also an experience of the present study. For example, *sharing* was both a subtheme of *facilitating* (in which facilitators sharing created a sense of equal status in the group between facilitators and participants), and also linked to an *active participation* (by which this process allowed participants to express themselves and make links with others). These subthemes interacted to contribute to the theme of *group collaboration,* illustrating a dynamic interaction of subthemes and themes in forming the in-the-moment processes within the group.

The field notes and observations of the three researchers who were present across all sessions, revealed that these dynamic processes appeared to take place more quickly in sessions 2 and 3; this observation was confirmed after viewing the video data. This may have been linked to the familiarity of the setting and as the group, including the facilitators, became more adept at creating an atmosphere that promoted *active participation*.

That there were no clear changes found in the frequency of codes or in the identified themes across the three sessions may be a limitation of the methodology used, or a reflection of the high level of engagement across the sessions, perhaps due to the different objects used. It is also possible that benefits were limited to in-the-moment changes and were not maintained or built on across sessions due to the range of impairments associated with the types of dementia the participants in this study were living with. Future research could explore this further by focussing on a greater number of sessions to see effects over time.

### Dementia care implications

This study offers a tentative understanding of the processes through which group object handling sessions may promote wellbeing in people living with dementia. Although this was a small-scale exploratory study, it nevertheless highlights key components of sessions that can inform future training of facilitators to optimise sessions for this population. This also has important implications for the role of museums in public health ( Camic & Chatterjee, 2013), and social prescribing opportunities ( Todd *et al*., 2017) for health service staff engaged in dementia care services. For example, in line with public health programming and social prescribing initiatives ( NHS England, 2019) professionals, such as clinical psychologists and occupational therapists, could train people working with dementia across community and non-healthcare settings, to increase the accessibility and specificity of museum object handling programmes. Such interventions speak to the person-centred approaches advocated by Kitwood (1997) that see and champion the person and their strengths and abilities, rather than focussing on the cognitive and behavioural changes and losses.

Expanding training beyond those who work in museum and heritage settings would raise awareness of dementia in the wider community. This could also enable people working in creative settings to adapt their ways of working to be more accessible for PLWD in line with The Prime Minister’s Challenge on Dementia ( Department of Health, 2012). For example, the museum in which the study took place already provided sessions for members of the public to handle and engage with museum objects. Further training specific to dementia and the arts was provided to museum staff by HZ and PC prior to the present study. Similar training at other heritage and non-heritage community sites could also be undertaken in order to emphasise important aspects of the intervention that may hold therapeutic benefits for PLWD, such as supporting object exploration, discovery and group interaction rather than prioritising providing information.

### Strengths, limitations and recommendations for future research

The small sample size can be viewed as both a strength and a limitation of the research. The participants were not representative of the demographic diversity of the population of PLWD across characteristics such as ethnicity, socioeconomic status and type and stage of dementia, thus limiting the applicability of the present study’s findings. Further, due to the sample size, the statistical analysis lacked power and the general lack of significant findings is possibly due to a Type II error. More longitudinal data collected over several months would be helpful to understand the ongoing effect of the sessions on subjective wellbeing. Input from care partners (spouse, family member, close friend) about their observations would further illuminate if the sessions were impactful in day-to-day life.

However, the small group size across multiple sessions, provided a unique opportunity to examine in-the-moment processes that have not yet been reported in previous studies within this population and therefore the findings offer an in-depth and comprehensive account of the sessions that took place. This also allowed each participant more time and space to participate and interact, which may be particularly important within the context of dementia related difficulties. Further multiple-session groups could also identify whether this has an impact on themes such as *active participation* and its implications for understanding the agency of people with dementia ( Van der Byl Williams, 2021; Zeilig *et al.,* 2019).

Our research supports what a carer in MacPherson *et al.* (2009) put so succinctly, “You do it for the moment encapsulates a sense that an activity is worthwhile even if it gives benefit only whilst running.” (p. 748), yet we also agree with Keady *et al*. (2020) that knowing more about what a moment is made up of, along with its antecedent and subsequent moments, will provide “greater conceptual and methodological” innovation about ‘moments’ so that they can be “positioned and linked together to provide a more holistic understanding of lived experience” (p. 18). Future studies would do well to explore what occurred in the ‘moments’ before museum object handling sessions (e.g. breakfast, leaving home, the journey to the museum) and in the ‘moments’ occurring after the sessions through interviews, diaries, mobile phone dictation recordings and/or visual maps. This can further help to contextualise and connect different moments in the lives of people living with dementia and provide deeper and better-informed understanding of their lived experiences ( Harding *et al.,* 2021).

In addition, we encourage future researchers to consider other qualitative methodologies to explore object handling interventions such as discourse analysis, narrative analysis and focused ethnography. 

There were important issues connected with recruitment. A wide net was cast to inform people about the study but recruiting a larger sample size proved surprisingly challenging for reasons that are not fully clear. The study required commitment to three sessions, with fixed dates and one location in central London. If sessions were held more frequently and were an open, drop-in style, rather than requiring commitment to specific dates, this may have increased recruitment. Future researchers need to consider building and improving *ongoing links* with services and charities that provide dementia care and support, such as those who advertised the present study, to build an atmosphere of trust to encourage more PLWD to engage in future heritage-based research. Engaging people with dementia and carers directly was a key aspect of this project but we may have underestimated the necessary time to do this.

This study focussed on the participation of people with a dementia diagnosis and did not include carers. For some, it may not be feasible to attend museum programmes without the participation of carers, whilst for others, doing an activity on their own may prove beneficial and enjoyable. Future research comparing the processes during object handling sessions with and without carers could be an interesting avenue to explore the themes identified here such as *active participation* and *group collaboration*, as carer’s participation in museum interventions for PLWD has been found to have both positive and negative impacts ( Kinsey *et al*., 2021). Likewise, giving participants a choice if they wanted PLWD-carer groups or preferred only PLWD groups would provide more opportunities for co-decision making. 

A strength of the present study was its ability to provide ecologically valid object handling sessions in a well-known museum, as an accessible community intervention. In line with this, the present study also benefitted from using in-the-moment non-intrusive methods of data collection, rather than relying on other methods such as post-session interviews. Future research maximising on such measures may allow the benefits of interventions to be more fully explored. For example, previous studies in other arts interventions for PLWD have utilised in-the-moment methods to explore verbal fluency ( Eekelaar *et al.,* 2012; Young *et al*., 2015) and to interpret responses such as stress and positive stimulation through physiological measures ( Thomas *et al*., 2018). For future qualitative research, the work of Keady *et al*. (2020) provides noteworthy considerations for what ‘in-the-moment’ means for those living with different dementias. 

## Conclusion

This was the first study to explore the process of facilitated small group object handling sessions involving people living with a dementia, in a museum setting, across multiple sessions. Findings suggest a positive influence of object handling on the subjective wellbeing in people living with dementia, and identified four key themes ( *facilitation, exploring objects, active participation,* and *group collaboration*) to help explain the possible processes present in the facilitation of sessions, the roles of material objects, and person-to-person interactions. Facilitators’ guidance created conditions within the group that led to group members to actively participate in expressing themselves (including voicing different opinions), leading to group cohesion and collaboration between participants and facilitators in sharing responsibility for the group and building on each other’s ideas to discover more about objects. Objects were explored from many different perspectives and provided a shared focus within the group through which these processes took place. These findings should be viewed tentatively due to the small sample size; however, they offer important insights concerning how to optimise sessions for this population. Future research using multiple groups and a more diverse sample can extend the present study’s findings but perhaps more importantly, other qualitative methodologies may provide additional information about the narrative and discourse that occurs in museum object handling groups. And finally, as a freely available measure that is non-obtrusive to use and easy to score, the Canterbury Wellbeing Scales, should be considered in community-based programmes for early to middles stages of different dementias.

## Data availability

### Underlying data

***Qualitative data.*** Full transcripts of group sessions are not publicly available due to concerns about data protection and confidentiality. If qualified researchers are interested in using transcript data from this study they are asked to contact the corresponding author ( p.camic@ucl.ac.uk) describing the nature of their interest and their qualifications, the intended use of the data, plans for obtaining ethical approval at their respective institution, and signing a confidentiality agreement to assure protection of data whilst in their possession. When ethical approval has been provided as evidenced by a signed letter from the ethics panel, transcript data will be transferred via an encrypted and password protected file. Access to transcripts is only permitted by the requesting researcher(s) and cannot be shared with others.

***Quantitative data.*** Zenodo: Subjective wellbeing in people living with dementia: exploring processes of multiple object handling sessions in a museum setting underlying quantitative data. http://doi.org/10.5281/zenodo.4715016 ( Strohmaier *et al.,* 2021b).

This project contains the following underlying data:

- Underlying quantitative data- Canterbury Wellbeing Scales data.xlsx

### Extended data

Zenodo: Canterbury Wellbeing Scales: directions and scales. http://doi.org/10.5281/zenodo.4063768 ( Camic *et al.*, 2020).

This project contains the following extended data:

- Canterbury Wellbeing Scales.pdf

Zenodo: Extended files for Subjective wellbeing in people living with dementia: Exploring processes of multiple object handling sessions in a museum setting. http://doi.org/10.5281/zenodo.4667498 ( Dickens *et al.,* 2021).

This project contains the following extended files:

- Protocol for object handling sessions.pdf
- Objects used in the study.pdf (Images and information about the material objects used in the study)
- Excerpt from a coded transcript.pdf
- Initial & Final codebooks.pdf ( Dickens *et al*., 2021).

Data are available under the terms of the Creative Commons Attribution 4.0 International license (CC-BY 4.0).
