# Supplementary material for: Subjective wellbeing in people living with dementia: exploring processes of multiple object handling sessions in a museum setting
Source: Wellcome Open Res. 2021 Jun 10;6:96. Originally published 2021 May 6. [Version 2] doi: 10.12688/wellcomeopenres.16819.2 (PMC8215562; doi:10.12688/wellcomeopenres.16819.2)
Supplement: Peer review history

# Peer review: Reviewer response for version 2

**DOI:** 10.21956/wellcomeopenres.18713.r44388

**Published:** 2021-06-18

**Authors:** Lee Burnside

**Affiliations:** 1 Division of Gerontology and Geriatrics, University of Washington, Seattle, WA, USA

I appreciate Dr. Camic et al. responding so thoroughly to my inquiries. This is a well written study, although small, that contributes significantly to the area of use of museums in promoting quality of life for persons with dementia.

Is the work clearly and accurately presented and does it cite the current literature?

Yes

If applicable, is the statistical analysis and its interpretation appropriate?

Yes

Are all the source data underlying the results available to ensure full reproducibility?

Yes

Is the study design appropriate and is the work technically sound?

Yes

Are the conclusions drawn adequately supported by the results?

Yes

Are sufficient details of methods and analysis provided to allow replication by others?

Partly

Reviewer Expertise:

Dementia, palliative care, museum studies

I confirm that I have read this submission and believe that I have an appropriate level of expertise to confirm that it is of an acceptable scientific standard.

# Peer review: Reviewer response for version 1

**DOI:** 10.21956/wellcomeopenres.18553.r43756

**Published:** 2021-05-21

**Authors:** Lee Burnside

**Affiliations:** 1 Division of Gerontology and Geriatrics, University of Washington, Seattle, WA, USA

This is a small mixed method study looking at the effect of object handling for persons living with dementia. It constituted four individuals diagnosed with dementia, participating in three exploratory sessions of one hour each. Sessions were led by a facilitator, audio and video recorded. The Canterburry Wellbing Scales were used pre and post experience.  A thematic map was developed from qualitative analysis of the sessions.

 Comments:

 First of all I would like to thank the authors for their contribution on expanding the effects of museum based interventions for persons with dementia. Current literature and the framework in which this study sits were well presented in the paper.

 My comments are mostly addressing clarifications.

 I would like to know more details regarding the process itself. How many objects were presented per session? How were they chosen? Page 6 what is the purpose behind “novel, diverse”, etc. Was one object discussed at a time among all participants as it was passed around? I even wonder how close participants were sitting to each other and the facilitator? (Pre Covid I am assuming). I would like to know more about the questions asked. Was there a process or script in which the questions were created? The second session with smell adds a little complexity, why were smells chosen? Was there also a tactile component such as touching the cinnamon sticks? A table with a list of the objects and attributes might be helpful.

 There is little data, to my knowledge, about how persons with dementia respond to the types of artwork presented in museum programs for dementia/care partners 9abstract, sculpture, everyday objects in new settings etc).  It appears this study could contribute to that literature with more description of participants specific reactions?

 I would like to see a little more written about the “museum setting”.  A conference room was used within the museum. Is there an aspect of one coming to a museum, a special place, that is specifically helpful to this process? Was it the use of museum objects that were special? I wasn’t sure if there was a connection to current displays that might tie in with which objects were presented?

 Minor comments:

 P4 What was the intervention in the Ander study?

 P4 Paddon, what objects were used?

 P4 The questions at the end of the paragraph referencing Keady seem confusing. Is the inference meant to be that these questions are less relevant?

 P5 Stakeholder involvement – not sure this paragraph is needed.

 With the thematic map I wondering about the overlap/connection with socialization and group collaboration as socialization tends to be a key factors in activities such as yours. Facilitators sharing, sharing responsibility and participants sharing, are these components of socialization? This is touched upon in summary of findings “shared focus” but again is this at least in part socialization?

 P14 discussion of the olfactory experience. I wonder if this and description of the objects could be earlier in the paper – methods.

 With the pandemic occurring and impacting in person gatherings and in person events for persons with dementia (even post Covid) I wonder if it would be helpful to postulate how this process may be done via video with participants having the same object in hand to discuss?

 Thank you for allowing to review this interesting paper.

Is the work clearly and accurately presented and does it cite the current literature?

Yes

If applicable, is the statistical analysis and its interpretation appropriate?

Yes

Are all the source data underlying the results available to ensure full reproducibility?

Yes

Is the study design appropriate and is the work technically sound?

Yes

Are the conclusions drawn adequately supported by the results?

Yes

Are sufficient details of methods and analysis provided to allow replication by others?

Partly

Reviewer Expertise:

Dementia, palliative care, museum studies

I confirm that I have read this submission and believe that I have an appropriate level of expertise to confirm that it is of an acceptable scientific standard, however I have significant reservations, as outlined above.

# Response

**Published:** 2021-06-04

**Authors:** Paul Camic

**Affiliations:** University College London, UK

**Responding to Reviewer 2, Dr. Lee Burnside**

 Thank you for your helpful and insightful comments and for your close read of our paper. Our responses to each of your comments follow. 

**1.**    I would like to know more details regarding the process itself. How many objects were presented per session? How were they chosen? Page 6 what is the purpose behind “novel, diverse”, etc. Was one object discussed at a time among all participants as it was passed around? I even wonder how close participants were sitting to each other and the facilitator? (Pre Covid I am assuming). I would like to know more about the questions asked. Was there a process or script in which the questions were created? 

**Response:** Thank you for these comments and questions. We have added to and revised the following (subsection Object handling sessions): “Facilitators welcomed participants at the beginning of each session passing around one object at a time and encouraged touching and generating discussion through asking a range of sample prompts and questions to encourage participation and exploration before sharing information about each object. Questions were used flexibly, in an open dialogic way, within the sessions based on the interaction of participants. A total of 20 objects were used across the sessions; 14 objects came from the museum’s collections including 4 spices, while others were contributed by PC. Six to nine objects were used in each session. Objects were jointly decided upon by the facilitators and researchers and selected to be novel and diverse in their cultural, historical and sensory qualities in order to maximize variety, interest and opportunities for exploration that did not rely on previous memories or knowledge. The study’s protocol, including sample questions, photos and descriptions of the objects used in each session are available as extended open access files (Dickens et al., 2021).

**2.  **  The second session with smell adds a little complexity, why were smells chosen? Was there also a tactile component such as touching the cinnamon sticks? A table with a list of the objects and attributes might be helpful. 

 R **esponse:** We chose to include smells based on (a) previous research about olfactory responses in dementia care (Burns et al., 2002; Jacob & Collier, 2014; Kitwood, 1997; Tischler & Clapp, 2020), and (b) the museum had a collection of spices that were frequently refreshed and part of the interactive visitor experience to relevant collections. The museum facilitators and researchers agreed that this would both highlight objects in the collection and provide an olfactory and tactile experience. Spices were contained in small glass bottles and passed around, one at a time, as were the other objects. This generated a good deal of discussion about how these spices might be used (e.g. in cooking and medicinally across cultures and in past times). 

 In the subsection, Subjective wellbeing  (in the Discussion section), we have added the following: “Olfactory experiences in dementia care (Burns et al., 2002) have generally revealed positive responses across different stimuli (e.g. Jacob & Collier, 2014; Tischler & Clapp, 2020) and should be considered when planning object handling sessions.”

**3.  **  There is little data, to my knowledge, about how persons with dementia respond to the types of artwork presented in museum programs for dementia/care partners (abstract, sculpture, everyday objects in new settings etc). It appears this study could contribute to that literature with more description of participants specific reactions? 

**Response:** We definitely agree, there is a paucity of research that looks specifically at how people with dementia respond to different types of art works. The Meet Me at MoMA programme was the first study that we are aware of that looked at figurative and abstract modern art, and although not designed as a comparative study, did find abstract art often produced more engaged and longer discussions. 

     A cross-sectional observational study, undertaken in The Netherlands, addresses this issue: Hendriks, I., Meiland, F., Slotwinska, K., Kroeze, R., Weinstein, H., Gerritsen, D., & Dröes, R. (2019). How do people with dementia respond to different types of art? An explorative study into interactive museum programs. International Psychogeriatrics, 31(6), 857-868. doi:10.1017/S1041610218001266. An earlier, smaller scale, mixed-methods study in the UK, also looked at art works in two different museums (contemporary and 17th-18thc, respectively), Camic, P. M., Tischler, V. and Pearman, C. H. (2014). Viewing and making art together: a multi-session art-gallery-based intervention for people with dementia and their carers. Aging & Mental Health, 18, 161–168. doi: 10.1080/ 13607863.2013.818101, and there may be others. 

     Because the present study used different types of material objects, most of which would not be considered “art”, we are not confident that describing individual responses in more detail than we have already done, would make a meaningful contribution. However, future researchers could access our audio and video files (access instructions are provided at the end of the article) and explore this interesting issue.

**4.  **  I would like to see a little more written about the “museum setting”. A conference room was used within the museum. Is there an aspect of one coming to a museum, a special place, that is specifically helpful to this process? Was it the use of museum objects that were special? I wasn’t sure if there was a connection to current displays that might tie in with which objects were presented? 

**Response**: In the Methods section (Design and setting subsection) we have made clear this is a “specialist” museum and added the following: “The museum’s focus is to connect science, medicine, health and the arts and this is reflected in its collections, special exhibitions, public engagement, and lecture programme. The object handing sessions took place in a well-lit, comfortable conference room with windows on a high floor. Facilitators and participants were seated around a rectangular table and close enough to allow for the easy passing of objects. The physical environment of the room was considered and the decision to hold the sessions in a private space rather than a gallery room was made to allow quiet for listening, fewer distractions to support engagement and confidentiality for audio recording.”  

**5. **   P4 What was the intervention in the Ander study?

**Response:** They used museum objects from London and Oxford museums in inpatient neurological rehabilitation (London), outpatient neurological rehabilitation (Oxford), and inpatient mental health care (Reading). Objects were brought to the respective location and occupational therapy clinicians engaged one-on-one with participants. Forty-two museum objects comprising specimens from anthropology, archaeology, art, geology and zoology collections were compiled and brought in boxes, each box contained 6 different objects. 

**6. **   P4 Paddon, what objects were used?

**Response:  **Objects comprised archaeological and ethnographic artefacts, etchings and printing plates, fossils, mineral samples and zoology specimens that varied in their tactile, visual and kinaesthetic properties (e.g. Egyptian bronze figurine, Neolithic hand axe, 1950s print, fossilized shark's tooth, piece of agate and turtle carapace).

**7.  **  P4 The questions at the end of the paragraph referencing Keady seem confusing. Is the inference meant to be that these questions are less relevant? 

**Response**: Apologies if we were not clear. We meant to imply that these questions are more relevant and have added the following (underlined): “More relevant questions for a dementia population include, How long do these moments last? What comes before and after them? Can different moments be linked together? ( Keady et al., 2020).”

**8.**    P5 Stakeholder involvement – not sure this paragraph is needed. 

**Response: **We feel it is relevant for the following reasons: Very few psychometric questionnaires have involved people with dementia in their development. Most often, researchers decide which questions are the most important to ask with the assumption that they know best. The Canterbury Wellbeing Scales (CWS) were developed with direct input from people with dementia and caregivers, along with older adult specialist psychologists and Alzheimer’s Society staff. Secondly, the length and structure of the sessions in the present study were based on feedback from participants (people living with a dementia and caregivers) in previous studies, consulting with members of the Rare Dementia Support charity (https://www.raredementiasupport.org/) and talking to museum staff at multiple musuems. In addition to these reasons, we concur with the (UK) Health Research Authority that states, “Public involvement in research means research that is done ‘with’ or ‘by’ the public, not 'to', 'about' or 'for' them. It means that patients or other people with relevant experience contribute to how research is designed, conducted and disseminated” (https://www.hra.nhs.uk/planning-and-improving-research/best-practice/public-involvement/ ). 

**9.  **  With the thematic map I wondering about the overlap/connection with socialization and group collaboration as socialization tends to be a key factors in activities such as yours. Facilitators sharing, sharing responsibility and participants sharing, are these components of socialization? This is touched upon in summary of findings “shared focus” but again is this at least in part socialization? 

**Response: **This is an insightful comment, and we agree socialisation is potentially part of what is described in the thematic map in that the sessions were formed by people socially interacting with others around a specific task and may have adapted to behave in a way acceptable to the group. How to socialise in a group setting is something most people likely learn in school at a young age. However, the thematic map describes and attempts to tease apart some of the processes associated with what is specific to object handling, and socialisation, for us, is an overly broad construct that would need to be further broken down and measured (e.g. specific behaviours, eye movements, tone of voice, reactions to verbal comments, etc.), in our opinion, to be useful. Considering that two people had a type of dementia that can make socialising quite challenging (FTD), the responses and interactions of participants across the three sessions provide support that object handling—as a focal activity—has some potential in dementia care.

**10.**    P14 discussion of the olfactory experience. I wonder if this and description of the objects could be earlier in the paper – methods. 

**Response:** Thank you for this suggestion. We have added the following sentence to the Methods section in the Object handing sessions, subsection: “The second session also involved smelling four spices contained in small glass bottles (passed one at a time to participants) that were part of the museum’s collection. Olfactory experiences in dementia care have been shown to be potentially engaging experiences that can evoke personal responses an reflection.”

**11.  **  With the pandemic occurring and impacting in person gatherings and in person events for persons with dementia (even post Covid) I wonder if it would be helpful to postulate how this process may be done via video with participants having the same object in hand to discuss? 

**Response:** Thank you for this. The first author is currently engaged with other colleagues as part of a funded study which examines personal objects within the context of on-line support group sessions with dementia caregivers who are caring for someone with a rare form of dementia. We feel the information we have to date, while promising, requires additional development. The study reported here was completed before the pandemic but it is interesting to ponder, however, about how object handling sessions may be done via video. It is could be important to explore this as a way to increase accessibility, but this would forgo important aspects of the museum setting (e.g. physically being around others, all handling the same objects including those you wouldn't usually get to touch as part of museum collection). 

        Facilitators could video from a carefully selected space in the museum (interesting but not too busy background that conveys the presence of a museum), orienting people to the room they can see on the screen and to each other, allowing for pauses after each person speaks for any delays and some time at the start for any technical issues to be problem solved. Facilitators might consider presenting visually some objects and generate discussion (although this would not be object handling) but may limit who can participate (sight/hearing/staying focused when on a screen and other things going on in the setting they are joining from) and may require the support of someone at home to navigate the technology. Participants could share objects from home or all be asked to bring something such as a spice so all have the same object to touch/smell/discuss. Items could be posted out beforehand but unsure how this would work in practice. Just some thoughts.

# Peer review: Reviewer response for version 1

**DOI:** 10.21956/wellcomeopenres.18553.r43754

**Published:** 2021-05-11

**Authors:** Anna Ullán

**Affiliations:** 1 Advanced Research Unit in Pediatric Psychology, Department of Social Psychology and Anthropology, University of Salamanca, Salamanca, Spain

The article “Subjective wellbeing in people living with dementia: exploring processes of multiple object handling sessions in a museum setting. Version 1” describes a study that expands on previous research and explores the potential processes underlying the effect of multiple small group sessions on handling objects, carried out in a museum. A mixed design was used to assess the subjective wellbeing of participants with dementia, using a quantitative self-report measurement, the Canterbury Wellbeing Scales (CWS). A thematic analysis of the transcripts of the previously recorded sessions and the observers' field-notes was also carried out.

 The article begins with a good contextualization of the research on the role of art-based interventions to improve the quality of life of people with dementia. The most relevant and recent research on the subject is mentioned and, after commenting on it, the basic hypothesis of the study is presented: that the sessions of handling objects in the museum will increase the wellbeing of participants with dementia. The method section describes the aspects relating both to the quantitative measures used and the qualitative approaches. The results of this study indicate an average increase in participants' wellbeing when comparing the measures taken before the sessions with those carried out afterwards, although this increase does not reach statistical significance.

 The topics and sub-topics identified through the analysis of the session transcriptions and the field-notes suggest that wellbeing may have increased through facilitation processes that could have empowered the participants by facilitating their self-expression. Among the most important aspects of the work, its authors highlight that it provides a greater understanding of the processes through which handling object activities in a museum can promote the wellbeing of people with dementia.

 The main limitation of the work, also acknowledged by the authors, is the small sample size of people with dementia participating in this study, four people, which considerably reduces the applicability possibilities of the study's findings. It is true that the small sample size allows an in-depth analysis of issues that would not be possible with a larger sample. Still, given the social relevance of the issue of caring for and improving the quality of life of people with dementia, the study lacks more people, which would provide the authors' findings with a more solid basis.

 Despite this limitation, the adequate conceptual and methodological basis of the work inclines me to recommend its publication. Subsequent studies may refer to this research and use a larger sample of participants with dementia.

Is the work clearly and accurately presented and does it cite the current literature?

Yes

If applicable, is the statistical analysis and its interpretation appropriate?

Yes

Are all the source data underlying the results available to ensure full reproducibility?

Yes

Is the study design appropriate and is the work technically sound?

Yes

Are the conclusions drawn adequately supported by the results?

Yes

Are sufficient details of methods and analysis provided to allow replication by others?

Yes

Reviewer Expertise:

Art Education for people with dementia

I confirm that I have read this submission and believe that I have an appropriate level of expertise to confirm that it is of an acceptable scientific standard.

# Response

**Published:** 2021-05-11

**Authors:** Paul Camic

**Affiliations:** University College London, UK

We would like to thank Dr Anna Ullán for taking the time to review our manuscript. We appreciate her comments and agree, a larger number of participants would have strengthened the study. Unfortunately, recruitment was challenging, perhaps due to the time of year (winter) but *possibly* due to other circumstances. These include the required commitment to come to three sessions, difficulty arranging transport from home to the museum in a busy part of central London, and any uncertainty people may have had about coming to a new place (the museum). The study also coincided with a dramatic reduction of funding for a charity we had partnered with. They had to close their local day and drop in facility, which was a close walk from the museum.
